# Peptide Receptor Radionuclide Therapy and clinical associations with renal and hematological toxicities and survival in patients with neuroendocrine tumors: an analysis from two U.S. medical centers

**DOI:** 10.1007/s00432-024-06020-w

**Published:** 2024-11-02

**Authors:** Tao Xu, Joseph S. Dillon, Mary A. Maluccio, Dawn E. Quelle, Sarah H. Nash, Hyunkeun Cho, Kristen E. Limbach, Nicholas J. Skill, Yvette Bren-Mattison, Michael A. O’Rorke

**Affiliations:** 1 Department of Epidemiology, College of Public Health, University of Iowa, Iowa City, IA USA; 2 ENETS Center of Excellence, Holden Comprehensive Cancer Center, University of Iowa, Iowa City, IA USA; 3 Department of Internal Medicine, Carver College of Medicine, University of Iowa, Iowa City, IA USA; 4 Department of Surgery, School of Medicine, Louisiana State University Health Sciences Center, New Orleans, LA USA; 5 Department of Neuroscience and Pharmacology, Carver College of Medicine, University of Iowa, Iowa City, IA USA; 6 Department of Biostatistics, College of Public Health, University of Iowa, Iowa City, IA USA; 7 Department of Surgery, School of Medicine, Tulane University, New Orleans, LA USA

**Keywords:** Neuroendocrine tumors, Peptide Receptor Radionuclide Therapy, Renal toxicity, Hematological toxicity, Survival

## Abstract

**Purpose:**

Renal and hematological toxicity are side effects and dose-limiting factors of Peptide Receptor Radionuclide Therapy (PRRT). We aimed to assess the changes in renal and hematological function and associations with survival in neuroendocrine tumor (NET) patients treated with PRRT.

**Methods:**

A retrospective cohort of 448 NET patients treated with either ^177^Lu-DOTATATE or ^90^Y-DOTATOC were followed for changes of renal and hematological function. Renal function was assessed by monitoring changes in serum creatinine, blood urea nitrogen and estimated glomerular filtration rate. Hematological function was determined by examining changes in white blood cell counts (WBC), platelet counts, and hemoglobin levels over time. Piecewise linear mixed effect models were applied to model the longitudinal repeated measurements of renal and hematological function. Overall survival (OS) and progression-free survival (PFS) were modelled using Cox proportional hazard regressions.

**Results:**

Of the 448 PRRT treated patients, 335 received ^177^Lu-DOTATATE (74.78%) and 113 were treated with ^90^Y-DOTATOC (25.22%). Comparing patients treated with ^177^Lu-DOTATATE to those treated with ^90^Y-DOTATOC, renal function did not differ significantly prior to, during or after PRRT. Compared with patients treated with ^90^Y-DOTATOC, significantly decreased indicators of hematological function were observed in those treated with ^177^Lu-DOTATATE prior to and during PRRT treatment (WBC: estimate, -0.10, 95% CI, -0.15 to -0.05; *P* < 0.001; platelet count: estimate, -2.53, 95% CI, -3.83 to -1.24; *P* < 0.001), and no significant recovery was observed in hematological function post PRRT. Individuals who received ^177^Lu-DOTATATE tended to have a longer PFS (hazard ratio, 0.47, 95%CI: 0.28–0.79, *P* = 0.004) compared with ^90^Y-DOTATOC, but there was no difference in OS.

**Conclusion:**

There was no significant renal, but minor hematological toxicity, in patients treated with ^177^Lu-DOTATATE compared with ^90^Y-DOTATOC. Compared to ^90^Y-DOTATOC, ^177^Lu-DOTATATE appears to enhance PFS, but not OS. Treatment with ^177^Lu-DOTATATE may necessitate follow-up for hematological toxicity irrespective of other therapies prior to PRRT.

**Supplementary Information:**

The online version contains supplementary material available at 10.1007/s00432-024-06020-w.

## Introduction

Neuroendocrine tumors (NETs) are a heterogeneous group of uncommon neoplasms arising from neuroendocrine cells, accounting for around 1% of all new cancers diagnosed in the USA (Yao et al. 2008; SEER 2015). The incidence of NETs continues to increase worldwide with a 4.5-fold increase between 1975 and 2019 in the USA (Wu et al. 2024). The 1-, 3-, 5-year overall survival rates for patients with NETs are 93.0%, 86.1%, and 80.1%, respectively (Wu et al. 2024).

For patients with progressive metastatic neuroendocrine tumors, peptide receptor radionuclide therapy (PRRT) with radiolabeled somatostatin analogs has been a therapeutic option for decades (Kwekkeboom et al. 2005b; Ćwikła et al. 2010; Bodei et al. 2011, 2013; Imhof et al. 2011). 90-yttrium (^90^Y)-DOTATOC and 177-lutetium (^177^Lu)-DOTATATE have been the most commonly used forms of PRRT over the past two decades. ^177^Lu-DOTATATE is the current standard of care in progressive gastroenteropancreatic NETs and was approved by the European Medicines Agency (2021) and the US Food and Drug Administration (2018).

PRRT can result in irradiation of and damage to the renal arteriolar-glomerular area and hematopoietic tissue (Moll et al. 2001; Valkema et al. 2005; Bergsma et al. 2016a). For this reason, prophylactic amino acid solutions are co-infused during the therapeutic radionuclide to prevent renal radiopeptide retention and decrease the radiation dose to the kidneys from PRRT (Bodei et al. 2013). PRRT can deliver continuous radiation over time. Hence, it is important to assess renal and hematological toxicity because of their effects on the pharmacokinetics of radiopharmaceuticals, treatment efficacy and safety. Additionally, evaluation of renal and hematological function enables timely interventions, addressing issues before they escalate into serious complications. (Sabet et al. 2014). To our knowledge, there is no large longitudinal dataset on PRRT-related renal and hematological toxicities in the USA. Regional differences in cancer care, such as healthcare utilization patterns, may vary between European countries and the USA, leading to different study results (Commonwealth 2019). While upfront PRRT has showed improved clinical outcomes compared to upfront chemotherapy or targeted therapy (Pusceddu et al. 2022), the optimal timing of PRRT, early initiation-related toxicities, and survival benefits have still not been thoroughly examined. We performed a multicenter, retrospective cohort study to assess the changes in renal and hematological function in NET patients who received PRRT. We hypothesized that the degree of renal and hematological function may differ from pretreatment to post-treatment follow-up, as evidenced by corresponding changes in biomarkers. Moreover, the impacts of renal and hematological toxicity may vary by the radioisotopes used. Additionally, we hypothesized that there may be differences in survival between the different types of PRRT utilized.

## Materials and methods

### Patients

A retrospective cohort study was conducted on 530 patients with metastatic NETs from University of Iowa Hospitals and Clinics (UIHC) and Louisiana State University (LSU) institutional datasets. We identified patients who received therapeutic ^90^Y-DOTATOC or ^177^Lu-DOTATATE from January 2001 to September 2022. Eligible subjects had: (1) histological diagnosis of a NET (any grade) and (2) received at least one cycle of ^177^Lu-DOTATATE or ^90^Y-DOTATOC for therapeutic purposes. In order to assess the long-term effects of PRRT, patients with one or fewer measurements of hematological and renal function were excluded (*N* = 28). In addition, patients who received a second PRRT course (i.e., PRRT re-treatment/salvage PRRT) after the first course of PRRT, or patients who received combinations of ^177^Lu-DOTATATE and ^90^Y-DOTATOC were excluded (*N* = 54). All PRRT infusions performed on patients were accompanied by prophylactic amino acid infusions. All patients provided informed consent for recruitment into the study. The consent form was designed to obtain broad and enduring consent for both retrospective and prospective use. This study was approved by the University of Iowa Institutional Review Board (IRB ID: 199911057) under a data use agreement with LSU and was conducted following the Declaration of Helsinki.

### Outcomes

Pre-PRRT screening was undertaken 2 weeks before each cycle and during treatment checks. Post-PRRT lab tests and markers including complete blood count with differential and renal function should be monitored at roughly 1 month, 3 months, 6 months and 12 months after treatment. Renal function was measured by serum creatinine (mg/dL), blood urea nitrogen (BUN) (mg/dL) and estimated glomerular filtration rate (eGFR) (mL/min/1.73m^2^). The level of eGFR was dichotomized into eGFR < 60 and eGFR ≥ 60 to align with the Common Terminology Criteria for Adverse Events (CTCAE) 5.0, grade 2 or higher chronic kidney disease (eGFR ≤ 59 − 30). Hematological function was measured by white blood cell count (WBC) (K/mm^3^), platelet count (K/mm^3^), and hemoglobin concentration (g/dL). We defined the time between the date of PRRT initiation and the first objective progression (local or metastatic) or death (whichever came sooner) as progression-free survival (PFS). Overall survival (OS) was defined as the time between the date of first PRRT infusion and death from any cause. In case of loss to follow-up or stable disease status, patients were censored at the time of their last clinic contact. The definition of “progression” includes tumor changes assessed by imaging techniques (computed tomography, magnetic resonance imaging, positron emission tomography, or ultrasound scans). Response Evaluation Criteria in Solid Tumors 1.1 were used to define progression on imaging. Parameters included over a 20% increase in the sum of diameters of the target lesions at the level of the local disease and metastases (with an absolute increase of at least 5 mm) or the appearance of one or more new lesions measured on anatomical imaging, or detection of new lesions by imaging of the same modality when subsequently performed. Diagnostic imaging was done at 1–3 months, 6 months, and 12 months after the completion of all treatment cycles and remaining follow-up evaluations were conducted every 3–12 months if clinically indicated or per primary care team.

### Covariates

Pre-PRRT clinicopathological data, including age at diagnosis (in years), sex at birth (male vs. female), race/ethnicity (non-Hispanic Black vs. non-Hispanic White vs. other), primary site of the tumor (gastrointestinal (GI) tract vs. pancreas vs. lung vs. other vs. unknown), World Health Organization (WHO) modified tumor grade (G1 vs. G2 vs. G3), surgical resection of the primary tumor (yes vs. no), liver metastases (yes vs. no), lymph nodes metastases (yes vs. no), bone metastases (yes vs. no), liver directed therapy prior to treatment (yes vs. no), type of PRRT (^177^Lu-DOTATATE only vs. ^90^Y-DOTATOC only), upfront chemotherapy or targeted therapy (see Supplementary Materials) (yes vs. no) were retrieved from the two institutions of primary care. Primary tumor sites in the GI tract included the esophagus, stomach, small intestine, large intestine, appendix and rectum. Lung NETs included tumors arising from the bronchus or lung. The number of comorbid conditions (i.e., Charlson Comorbidities) (Charlson et al. 1987) was counted and classified into three categories (0 conditions vs. 1–2 conditions vs. 3 or more conditions). A variable was used to account for the variation in date of last contact and measurements between the two institutions of primary care (UIHC vs. LSU). The number of PRRT administrations was categorized as < 3 cycles vs. 3–4 cycles. The time between date of diagnosis and first cycle of PRRT was calculated (in years) to account for the timing of PRRT and the secular trend of ^177^Lu-DOTATATE approval.

### Statistical analysis

Categorical variables were summarized by counts (percentages) and continuous variables by means (standard deviations). Piecewise linear mixed models were used because of the occurrence of repeated measurements of renal and hematological function within each individual. We used continuous time variables to capture all relevant data points, rather than fixed time intervals, to account for lab tests occurring outside of these exact intervals. We defined the follow-up time prior to and during PRRT cycles as Time 1, and the follow-up time after the last cycle of PRRT as Time 2. Time 1, Time 2 and type of PRRT were fixed effect terms. To account for within-subject correlations, we applied three random components, including a random intercept and two random slopes. Interaction terms (i.e., Time 1 × type of PRRT, Time 2 × type of PRRT) were added when assessing changes in toxicity indicators before or after treatment for between- and within-group (i.e., ^177^Lu-DOTATATE and ^90^Y-DOTATOC) comparisons. Interaction terms were used to determine whether changes of renal and hematological function differed not only by different types of radiopharmaceuticals but also due to time. Relevant assumptions (i.e., Schoenfeld residuals for proportional hazards, normality of residuals and linearity of predictors) for the modeling were tested. Kaplan-Meier survival curves were generated for PFS and OS, and the log-rank test was applied to compare survival distributions. We applied the multivariable Cox proportional hazards model for PFS after assessment of the proportional hazards assumption. For OS, we applied a Weibull accelerated failure time model, a parametric model that provides an alternative to the commonly used proportional hazards model. Sensitivity analyses included: (1) evaluations of the dichotomized grade 2 or 3 renal and hematological toxicities, including increased creatinine, decreased WBC, decreased platelet count, and anemia (decreased hemoglobin) according to the CTCAE 5.0; (2) a bivariate analysis comparing the PRRT patients with or without upfront chemotherapy or targeted therapy; (3) a subgroup analysis of changes in hematological function in UIHC patients who received ^177^Lu-DOTATATE considering chemotherapy after PRRT (i.e., time-varying covariate). A P-value of < 0.05 was considered statistically significant. Statistical analyses were performed using R (R Foundation, Vienna, Austria) and SAS 9.4 (SAS Institute, Cary NC, USA).

## Results

The demographics and clinicopathological characteristics of 448 eligible NET patients who received PRRT are shown in Table 1. Of the 448 patients, 335 were treated with ^177^Lu-DOTATATE PRRT (74.78%) and 113 were treated with ^90^Y-DOTATOC (25.22%). Male patients outnumbered females (56.79% vs. 43.23%). The median post PRRT follow-up for renal and hematological function was 8.83 months (range 0.1–180 months). There were no differences of post PRRT follow-up between ^177^Lu-DOTATATE and ^90^Y-DOTATOC groups (*P* > 0.05). The average total administered activity at the individual level for patients who received ^177^Lu-DOTATATE was higher (642.33 mCi [range 100–814 mCi]) compared to that of patients who received ^90^Y-DOTATOC (340.97 mCi [range 120–600 mCi]). Most patients (77.63%) received PRRT and follow-up care within the USA at two institutions (UIHC and LSU).

**Table 1:** Characteristics of patients who received PRRT

	All (*N* = 448)	^177^Lu-DOTATATE (*N* = 335)	^90^Y-DOTATOC (*N* = 113)
**Age at diagnosis (years)**, Mean (SD)	54.26 (13.70)	56.62 (12.48)	47.25 (14.78)
**Race/ethnicity**			
Non-Hispanic White	392 (87.50)	287 (85.67)	105 (92.92)
Non-Hispanic Black	37 (8.26)	33 (9.85)	4 (3.54)
Other	19 (4.24)	15 (4.48)	4 (3.54)
**Sex at birth**			
Male	250 (55.80)	182 (54.33)	68 (60.18)
Female	198 (44.20)	153 (45.67)	45 (39.82)
**Primary tumor site**			
GI tract	242 (54.20)	203 (60.60)	39 (34.51)
Pancreas	106 (23.66)	67 (20.00)	39 (34.51)
Lung	31 (6.92)	22 (6.57)	9 (7.97)
Other	33 (7.37)	21 (6.27)	12 (10.62)
Unknown	36 (8.04)	22 (6.57)	14 (34.15)
**Upfront chemotherapy or targeted therapy (Yes)**	181 (40.40)	144 (42.99)	37 (32.74)
**WHO grade**			
G1	108 (24.11)	99 (29.55)	9 (7.96)
G2	184 (41.07)	154 (45.97)	30 (26.55)
G3	33 (7.37)	31 (9.25)	2 (1.77)
Unknown	123 (27.46)	51 (15.22)	72 (63.72)
**Liver directed therapy prior to PRRT (Yes)**	184 (41.82)	135 (41.28)	49 (43.36)
**Primary tumor resection (Yes)**	334 (74.89)	261 (78.38)	73 (64.60)
**Death (Yes)**	221 (49.33)	140 (41.79)	81 (71.68)
**Comorbidity**			
Myocardial infarction	3 (0.80)	1 (0.31)	2 (3.57)
Congestive heart failure	11 (2.92)	9 (2.82)	2 (3.57)
Peripheral vascular disease	9 (2.39)	5 (1.56)	4 (7.14)
Cerebrovascular disease	6 (1.59)	2 (0.62)	4 (7.14)
Dementia	1 (0.27)	1 (0.31)	0
Chronic pulmonary disease	14 (3.71)	12 (3.74)	2 (3.57)
Rheumatic disease	1 (0.27)	1 (0.31)	0
Peptic ulcer disease	3 (0.80)	1 (0.31)	2 (3.57)
Mild liver disease	41 (10.88)	25 (7.79)	16 (28.57)
Diabetes without chronic complication	33 (8.75)	26 (8.10)	7 (12.50)
Diabetes with chronic complication	10 (2.65)	8 (2.49)	2 (3.57)
Hemiplegia or paraplegia	1 (0.27)	0	1 (1.79)
Renal disease	20 (5.31)	17 (5.30)	3 (5.36)
Moderate or severe liver disease	11 (2.92)	6 (1.87)	5 (8.93)
Leukemia or lymphoma	3 (0.80)	2 (0.62)	1 (1.79)
**Count of comorbid conditions**			
0 conditions	272 (72.15)	245 (76.32)	27 (48.21)
1–2 conditions	91 (24.14)	66 (44.64)	25 (44.64)
3 or more conditions	14 (3.71)	10 (3.12)	4 (7.14)
**Liver metastases (Yes)**	402 (89.73)	305 (91.04)	97 (85.84)
**Lymph node metastases (Yes)**	272 (60.85)	209 (62.57)	63 (55.75)
**Bone metastases (Yes)**	122 (27.23)	90 (26.87)	32 (28.32)
**Number of PRRT administrations**			
<3 cycles	128 (28.70)	75 (22.52)	53 (46.09)
3–4 cycles	318 (71.30)	258 (77.48)	60 (53.10)
**Institution of primary care**			
UIHC	290 (64.73)	177 (52.84)	113 (100.00)
LSU	158 (35.27)	158 (47.16)	0
**Average administered activity per cycle (mCi) [GBq]**, Mean (SD)	182.55 (31.84) [6.75 (1.18)]	195.19 (16.08) [7.22 (0.59)]	145.10 (37.16) [5.37 (1.37)]
**Average total administered activity per individual (mCi) [GBq]**,** Mean (SD)**	565.96 (233.80) [20.94 (8.65)]	642.33 (219.62) [23.77 (8.13)]	340.97 (76.11) [12.61 (2.82)]
**Number of weeks between cycles**, Mean (SD)	9.62 (8.49)	9.71 (5.24)	9.38 (14.11)
**Reduced radioactivity (Yes)**	52 (11.82)	35 (10.64)	17 (15.32)
**Location of receiving PRRT**			
Bad Berka, Germany	4 (0.89)	4 (1.19)	0
Basel, Switzerland	78 (17.45)	31 (1.19)	47 (41.96)
Rotterdam, Holland	6 (1.34)	6 (1.79)	0
Houston, Texas, USA	5 (1.11)	5 (1.49)	0
Kansas City, Missouri, USA	2 (0.45)	2 (0.60)	0
LSU	156 (34.90)	156 (46.57)	0
London Ontario, Canada	1 (0.22)	1 (0.30)	0
Michigan, USA	2 (0.45)	2 (0.60)	0
Montefiore, Italy	1 (0.22)	1 (0.30)	0
NIH, USA	1 (0.22)	1 (0.30)	0
UIHC	191 (42.73)	126 (37.61)	65 (58.04)
**Time between date of diagnosis and initiation of PRRT (years)**, Mean (SD)	6.59 (5.72)	6.81 (5.98)	5.95 (4.84)

Categorical variables were summarized by counts (percentages) and continuous variables by mean (standard deviation [SD]).

### Renal function

The longitudinal changes in renal function from multivariable linear mixed effect models are shown in Table 2. From the pretreatment to the PRRT course (Time 1), change in renal function did not significantly differ between ^177^Lu-DOTATATE and ^90^Y-DOTATOC patients (creatinine: estimate, 0.004, 95% CI, -0.003 to 0.01; *P* = 0.29; BUN: estimate, -0.02, 95% CI, -0.14 to 0.10; *P* = 0.78; eGFR: OR, 0.94, 95% CI, 0.87-1.00; *P* = 0.07). Likewise, after the last cycle of PRRT (Time 2), renal function remained stable over time for both ^177^Lu-DOTATATE and ^90^Y-DOTATOC patients (creatinine: estimate, 0.002, 95% CI, -0.004 to 0.01; *P* = 0.51; BUN: estimate, 0.15, 95% CI, -0.11 to 0.41; *P* = 0.26; eGFR: OR, 1.02, 95% CI, 0.95 to 1.10; *P* = 0.51).

During the whole study period, regardless of the type of PRRT utilized, older age at diagnosis was significantly associated with poorer renal function (*P* < 0.05) (Supplementary Table 1). In addition, female patients tended to have a significantly lower serum creatinine and BUN concentration than their male counterparts (*P* < 0.05). Compared with other sites of NETs, patients with GI-NETs or pancreatic NETs had a lower BUN concentration (GI-NETS: estimate, -3.00, 95% CI, -5.12 to -0.87; *P* = 0.01; pancreatic NETs: estimate, -3.00, 95% CI, -5.30 to -0.70; *P* = 0.006). Compared with patients with no comorbidities, patients with 1–2 comorbidities had higher BUN (estimate, 2.05, 95% CI, 0.39 to 3.70; *P* = 0.02), and patients with 3 or more comorbidities had the highest BUN of these groups (estimate, 3.47, 95% CI, 0.36 to 6.58; *P* = 0.03). A longer time interval between the date of diagnosis and initiation of PRRT was significantly associated with worse renal function (*P* < 0.05).

**Table 2:** Longitudinal changes in renal function

	Creatinine β_−adjusted_ (95% CI)	*P*	BUN β-_adjusted_ (95% CI)	*P*	eGFR (< 60) Odds ratio (95% CI)	*P*
Monthly rate of change in renal function prior to and during PRRT in patients who received ^90^Y-DOTATOC	-0.002 (-0.01, 0.01)	0.54	0.01 (-0.10, 0.11)	0.88	**1.11** **(1.03**,** 1.19)**	**0.01**
Monthly rate of change in renal function prior to and during PRRT in patients who received ^177^Lu-DOTATATE	0.002 (-0.002, 0.01)	0.28	-0.01 (-0.07, 0.05)	0.77	1.04 (0.99, 1.08)	0.12
Comparison of monthly rate of change prior to and during PRRT between ^177^Lu-DOTATATE and ^90^Y-DOTATOC (Time 1 × type of PRRT)	0.004 (-0.003, 0.01)	0.29	-0.02 (-0.14, 0.10)	0.78	0.94 (0.87, 1.00)	0.07
Monthly rate of change in renal function after last cycle of PRRT in patients who received ^90^Y-DOTATOC	0.004 (-0.002, 0.01)	0.23	0.05 (-0.19, 0.28)	0.69	0.97 (0.91, 1.04)	0.42
Monthly rate of change in renal function after last cycle of PRRT in patients who received ^177^Lu-DOTATATE	**0.006** **(0.003**,** 0.01)**	**< 0.001**	**0.196** **(0.08**,** 0.31)**	**< 0.001**	1.00 (0.94, 1.06)	0.87
Comparison of monthly rate of change after last cycle of PRRT between ^177^Lu-DOTATATE and ^90^Y-DOTATOC (addition of two interaction terms)	0.002 (-0.004, 0.01)	0.51	0.15 (-0.11, 0.41)	0.26	1.02 (0.95, 1.10)	0.51
Time between date of diagnosis and initiation of PRRT (years)	**0.02** **(0.01**,** 0.03)**	**0.001**	**0.29** **(0.17**,** 0.42)**	**< 0.001**	**1.41** **(1.23**,** 1.63)**	**< 0.001**

* Time 1 represents the follow-up time prior to and during PRRT

† Time 2 represents the follow-up time after the last cycle of PRRT

Age at diagnosis, type of PRRT, Time 1, Time2, Time 1 × type of PRRT, Time 2 × type of PRRT, race/ethnicity, sex at birth, primary tumor site, primary tumor resection, upfront chemotherapy or targeted therapy, count of comorbid conditions, number of PRRT cycles, time between date of diagnosis and initiation of PRRT, and institution were included in models

### Hematological function

The longitudinal changes of WBC, platelet count, and hemoglobin are shown in Table 3. In the period prior to and during PRRT (Time 1) comparing ^177^Lu-DOTATATE versus ^90^Y-DOTATOC, decreased levels of hematological function were observed (WBC: estimate, -0.10, 95% CI, -0.15 to -0.04; *P* < 0.001; platelet count: estimate, -2.46, 95% CI, -3.72 to -1.19; *P* < 0.001). However, there were no significant changes during post-treatment follow-up (Time 2) between ^177^Lu-DOTATATE and ^90^Y-DOTATOC in hematological function (WBC: estimate, -0.002, 95% CI, -0.04 to 0.04; *P* = 0.89; platelet count: estimate, 0.13, 95% CI, -0.99 to 1.24; *P* = 0.82; hemoglobin: estimate, 0.003, 95% CI, -0.03 to 0.04; *P* = 0.90). Minor monthly decreases in WBC, platelet counts, and hemoglobin were confirmed examining patients who received ^177^Lu-DOTATATE only in UIHC from the pretreatment through the PRRT course (Table 3).

**Table 3:** Longitudinal changes in hematological function

	WBC β_−adjusted_ (95% CI)	*P*	Platelet count β-_adjusted_ (95% CI)	*P*	Hemoglobin β-_adjusted_ (95% CI)	*P*
Monthly rate of change in hematological function prior to and during PRRT in patients who received ^90^Y-DOTATOC	-0.02 (-0.06, 0.02)	0.37	-0.25 (-1.37, 0.87)	0.66	**-0.01** **(-0.04**,** 0.01)**	0.34
Monthly rate of change in hematological function prior to and during PRRT in patients who received ^177^Lu-DOTATATE	**-0.12** **(-0.14**,** -0.09)**	**< 0.001**	**-2.71** **(-3.30**,** -2.11)**	**< 0.001**	-0.04 (-0.06, -0.03)	**< 0.001**
Comparison of monthly rate of change prior to and during PRRT between ^177^Lu-DOTATATE and ^90^Y-DOTATOC (Time 1 × type of PRRT)	**-0.10** **(-0.15**,** -0.04)**	**< 0.001**	**-2.46** **(-3.72**,** -1.19)**	**< 0.001**	-0.03 (-0.06, 0.001)	0.06
Monthly rate of change in hematological function after last cycle of PRRT in patients who received ^90^Y-DOTATOC	0.02 (-0.01, 0.06)	0.22	-0.15 (-1.15, 0.84)	0.76	-0.03 (-0.06, 0.001)	0.06
Monthly rate of change in hematological function after last cycle of PRRT in patients who received ^177^Lu-DOTATATE	0.02 (0.001, 0.04)	0.06	-0.02 (-0.52, 0.47)	0.92	**-0.03** **(-0.04**,** -0.01)**	**< 0.001**
Comparison of monthly rate of change after last cycle of PRRT between ^177^Lu-DOTATATE and ^90^Y-DOTATOC (addition of two interaction terms)	-0.002 (-0.04, 0.04)	0.89	0.13 (-0.99, 1.24)	0.82	0.003 (-0.03, 0.04)	0.90
Time between date of diagnosis and initiation of PRRT (years)	0.004 (-0.03, 0.04)	0.83	**-1.84** **(-3.53**,** -0.16)**	**0.03**	**-0.06** **(-0.09**,** -0.03)**	**< 0.001**

* Time 1 represents the follow-up time prior to and during PRRT

† Time 2 represents the follow-up time after the last cycle of PRRT

Age at diagnosis, type of PRRT, Time 1, Time2, Time 1 × type of PRRT, Time 2 × type of PRRT, race/ethnicity, sex at birth, primary tumor site, primary tumor resection, upfront chemotherapy or targeted therapy, count of comorbid conditions, number of PRRT cycles, time between date of diagnosis and initiation of PRRT, bone metastases and institution were included in models

Other differences during the study period in all patients treated with PRRT were as follows (Supplementary Table 2). Patients with older age at diagnosis tended to have lower platelet counts and hemoglobin levels (*P* < 0.05). The level of WBC was different among primary tumor sites (*P* < 0.05). Compared with non-Hispanic White patients who received PRRT, non-Hispanic Black and other racial groups had lower levels of WBC and hemoglobin. Patients with upfront chemotherapy or targeted therapy had lower levels of hemoglobin (estimate, -0.56, 95% CI, -0.92 to -0.20; *P* = 0.003) and platelet counts (estimate, -24.00, 95% CI, -43.53 to -5.87; *P* = 0.003) than those without chemotherapy exposure. Patients with a longer time interval between date of diagnosis and initiation of PRRT had decreased levels of platelets (estimate, -1.73, 95% CI, -3.43 to -0.04; *P* = 0.05) and hemoglobin (estimate, -0.06, 95% CI, -0.09 to -0.03; *P* < 0.001). In our cohort, one patient (0.3%) treated with ^177^Lu-DOTATATE (and pre-PRRT chemotherapy) was diagnosed with myelodysplastic syndrome (MDS) during post-treatment follow-up (time between last cycle of PRRT and date of MDS diagnosis was 1,399 days). No patients were diagnosed with acute myeloid leukemia.

### Survival analysis

**Fig. 1:**
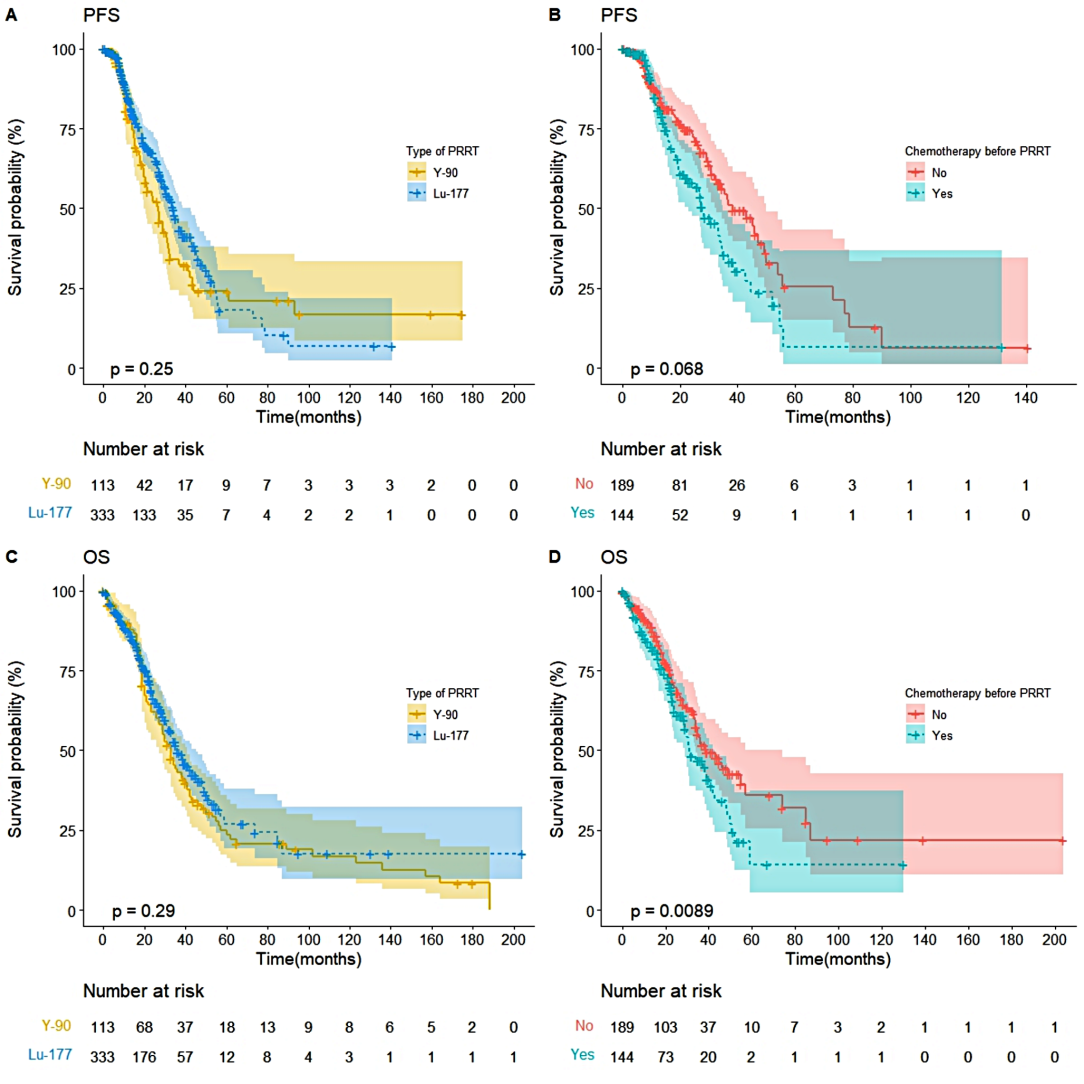
Overall survival (OS) and Progression-free survival (PFS). Kaplan-Meier curves of PFS (**A**) and OS (**C**) in patients treated with ^177^Lu-DOTATATE and ^90^Y-DOTATOC. Kaplan-Meier curves of PFS (**B**) and OS (**D**) in patients treated with ^177^Lu-DOTATATE only and chemotherapy before PRRT

The median OS was 36 months (95%CI: 33–45 months), and median PFS was 34 months (95%CI: 30–43 months) for individuals who received ^177^Lu-DOTATATE. The median OS was 33 months (95%CI: 27–41 months), and median PFS was 27 months (95%CI: 20–32 months) for individuals who received ^90^Y-DOTATOC. In the ^177^Lu-DOTATATE subgroup (Fig. 1B and D), upfront chemotherapy or targeted therapy was significantly associated with lower OS (*P* = 0.009), but not PFS (*P* > 0.05). In the UIHC ^177^Lu-DOTATATE sub-cohort (Supplementary Fig. 2), patients who received post-PRRT chemotherapy were more likely to have a better OS (*P* = 0.004).

In all patients, multivariable survival analysis showed that older individuals (HR, 1.03, 95%CI: 1.01–1.04, *P* < 0.001) and individuals who had upfront chemotherapy (HR, 1.75, 95%CI: 1.17–2.61, *P* = 0.01) had a lower OS (Supplementary Table 4). Additionally, NET patients who received 3–4 cycles of PRRT had a better OS compared to those who received less than 3 cycles of PRRT (HR, 0.27, 95%CI: 0.18–0.41, *P* < 0.001). Individuals who received ^177^Lu-DOTATATE tended to have a longer PFS (HR, 0.45, 95%CI: 0.26–0.76, *P* = 0.003) but not a longer OS (HR, 0.73, 95%CI: 0.44–1.21, *P* = 0.23) than patients who received ^90^Y-DOTATOC. Patients with pancreatic NETs, a higher tumor grade and inoperable disease were more likely to have upfront chemotherapy (*P* < 0.001) (Supplementary Table 5).

## Discussion

In this retrospective cohort study, we used longitudinal measurements to evaluate the renal and hematological toxicities and survival of patients treated with ^177^Lu-DOTATATE or ^90^Y-DOTATOC. Renal function was stable in all patients regardless of which type of PRRT they received from the pretreatment to the post-treatment periods. We observed decreased levels of hematological function during the pretreatment and PRRT course in patients who received ^177^Lu-DOTATATE. Patients who received ^177^Lu-DOTATATE had a better PFS but not OS compared to patients who received ^90^Y-DOTATOC. Older age at diagnosis, chemotherapy prior to PRRT, and fewer cycles of PRRT were related to worse OS.

### Renal toxicity

Our study demonstrated that renal function was relatively stable from pre-PRRT (Time 1) to post PRRT (Time 2) in patients who received either ^177^Lu-DOTATATE or ^90^Y-DOTATOC. Patients treated with ^90^Y-DOTATOC had lower administered activity than patients treated with ^177^Lu-DOTATATE. Our results are consistent with the majority of prior studies showing that the risk of renal toxicity from PRRT is low (Bergsma et al. 2016a; Duan et al. 2022; Puliani et al. 2022). However, conflicting associations have also been reported. For example, several studies of ^90^Y-DOTATOC have observed that 6.5–14% of patients developed grade 4 or 5 permanent renal toxicity (Imhof et al. 2011; Marincek et al. 2013; Romer et al. 2014). Up to 60% of patients treated with ^90^Y-DOTATOC only without amino acid-based protection reported renal toxicity (Marincek et al. 2013). Previous studies of ^177^Lu-DOTATATE reported that 0.6–1.5% of patients developed grade 3 or 4 renal toxicity (Kwekkeboom et al. 2005a, 2008; Bodei et al. 2009; Sabet et al. 2014; Bergsma et al. 2016a). In the pivotal NETTER-1 trial, 5% of patients treated with ^177^Lu-DOTATATE experienced grade 3 or worse renal toxicity and 1% of patients experienced grade 3 increased serum creatinine during the therapy, but no additional grade 3 or worse nephrotoxicity during long-term follow-up (Strosberg et al. 2021). The incidence of renal toxicity, represented by serum creatinine or GFR, was lower in patients treated with ^177^Lu-DOTATATE than that of ^90^Y-DOTATOC (Bodei et al. 2015). In addition to amino acid infusions, the higher radiation tolerability of ^177^Lu-DOTATATE than ^90^Y-DOTATOC is associated with a more non-uniform absorbed dose (Cremonesi et al. 2018). The heterogeneities of the sampling frame (e.g., stringent inclusion and exclusion criteria for clinical trials), included sample sizes, and primary tumor locations may cause variation in previous findings. Overall, our longitudinal analysis demonstrates (irrespective of radioligand therapy) that PRRT had minimal impacts on renal function with amino acid-based protection.

### Hematological toxicity

In general, we found that hematological toxicity from either ^177^Lu-DOTATATE or ^90^Y-DOTATOC PRRT was mild and acceptable during the therapy period (Time 1). We observed no apparent difference in recovery post PRRT (Time 2) comparing ^177^Lu-DOTATATE to ^90^Y-DOTATOC. Of note is that a higher proportion of individuals who received pre-PRRT chemotherapy in the ^177^Lu-DOTATATE group and a longer time interval between date of diagnosis and PRRT initiation, which may impact the comparison. Although we adjusted for these covariates, residual confounding may affect the explanation of the change of hematological function. Consistent with a previous study, our findings have shown decreases in hematological function during PRRT cycles (Bergsma et al. 2016). Between 5% and 13% of patients who received PRRT have been reported to develop grade 3 or 4 hematological toxicity (Kwekkeboom et al. 2008; Imhof et al. 2011; Pfeifer et al. 2011; Gupta et al. 2012; Sabet et al. 2013; Delpassand et al. 2014; Bodei et al. 2015; Bergsma et al. 2016a). Modest reversible hematological toxicity has been observed in previous studies among patients who received PRRT combined with chemotherapy (Bodei et al. 2009; Frilling et al. 2014; Kesavan et al. 2014). A progressive reduction in hematological function has been observed with ^177^Lu-DOTATATE therapy, which only recovered to baseline values within 24-months post-PRRT follow-up (Bodei et al. 2011). In our study, no significant improvements in hematological function following ^177^Lu-DOTATATE may be due to patients with longer follow-up and OS being more likely to receive post-PRRT chemotherapy. We also observed that patients with more comorbid conditions had a lower hemoglobin level. Similarly, a previous clinical trial indicated a mild residual anemia during post-PRRT follow-up, suggesting correlations between decreased hemoglobin levels and their existing chronic conditions (Bodei et al. 2011).

Serious hematologic toxicity was rarely reported in our cohort. In previous studies, therapy-related MDS and acute myeloid leukemia were found in patients treated with either ^177^Lu-DOTATATE or ^90^Y-DOTATOC (Otte et al. 1999; Kwekkeboom et al. 2003; Valkema et al. 2003; Strosberg et al. 2021; Kennedy et al. 2022). In the NETTER-1 trial, 2% of NET patients treated with ^177^Lu-DOTATATE developed MDS after receiving PRRT (Strosberg et al. 2021). Similar to previous studies, our results showed a less than 3% incidence of MDS (Sabet et al. 2013; Bodei et al. 2015; Brabander et al. 2017; Strosberg et al. 2021). The time to development of MDS in our study was 1,399 days, similar to prior reported median time between PRRT and MDS diagnosis of 1,351 days (Bodei et al. 2015). Therapy-related MDS and acute myeloid leukemia may be associated with mutational events induced by cytotoxic therapies, and the latency period ranges from a few months to decades (Godley and Larson 2008). Given the relatively short follow up in our study there may be more patients developing MDS if follow-up time was extended. Because of the limited prognosis of patients with metastatic NETs, the risk of these late hematological side effects has been considered relatively low (Sabet et al. 2013). In general, we may need to monitor modest PRRT-related hematological toxicities during PRRT therapies, particularly among patients with multiple comorbidities or a history of upfront chemotherapy or targeted therapy.

### Survival analysis

We observed neither OS nor PFS were affected by the time between date of diagnosis and initiation of PRRT. A recent study reported that the use of upfront PRRT after disease progression may be beneficial for longer PFS compared with upfront chemotherapy or targeted therapy (Pusceddu et al. 2022). Patients in our cohort received PRRT relatively late after diagnosis if they underwent chemotherapy or liver directed therapy first. Yet, early initiation of PRRT may have lower renal and hematological toxicities. Existing empirical evidence indicates that an earlier initiation/sequencing of ^177^Lu-DOTATATE is preferred before further disease development (Kwekkeboom et al. 2008). Delayed PRRT may expose patients to the progression of the underlying disease and cumulative toxicities of prior therapies, leading to additional renal impairment. However, no study has examined the optimal timing for PRRT in patients with NETs. Further research is needed to evaluate and optimize the strategy and sequence of systemic therapies, particularly given the increasing availability of PRRT.

We observed that patients who received 3–4 cycles of PRRT tended to have a better OS but not PFS compared with those treated with fewer cycles. Our results also showed that 3–4 cycles of PRRT were not associated with either renal or hematological toxicity. This could be explained by the good tolerability, but not tumor response, for patients treated with more PRRT cycles. Because of the absorbed dose to the kidneys per unit activity, the fractionation into number of cycles is more advantageous for ^90^Y-DOTATOC than for ^177^Lu-DOTATATE (Cremonesi et al. 2018). In current clinical practice, the scheduled four cycles of ^177^Lu-DOTATATE are recommended to ameliorate the increased risk of bone marrow toxicity (Hope et al. 2019).We also observed that patients receiving chemotherapy prior to PRRT did not have longer survival time compared with those without prior chemotherapy. Given that combination therapy of PRRT and chemotherapy was considered after the failure of chemotherapy or PRRT monotherapy (Yordanova et al. 2019), the shorter OS may represent increased aggressiveness of tumors in patients treated with PRRT and previous chemotherapy.

### Limitations

This study has several limitations. First, our retrospective cohort was composed of NET patients who only received PRRT. Compared with a randomized comparison design or matched non-PRRT controls, an assessment of the therapeutic effectiveness and tumor response to PRRT was not possible. However, the selection of all PRRT-treated patients is more relevant, because non-PRRT patients are likely to have less advanced disease (i.e., of questionable comparability). In addition, the post-PRRT follow-up period may not be sufficient to detect late toxicities and bone marrow toxicities. In contrast, we were able to observe early toxic events and subtle changes in lab values during the current follow-up period. The creatinine clearance loss, a more reliable indicator of renal function, could not be used in this study population due to a lack of information on the corresponding body weight at each time point assessed. Instead, we directly monitored the serum creatinine level repeatedly and kept all data points, which is acceptable to model changes in renal function over time. Additionally, the calculation formulas of eGFR were not available because of the long study time frame and the retrospective data extraction of electronic health records. Although all patients had renal protection during the PRRT, we lacked sufficient records on the specific formulas of the amino acid infusions. Multiple comparisons can lead to false positive results. However, the main conclusions were not distorted after applying Bonferroni corrected P values (0.05 divided by the 8 possible comparisons).

## Conclusion

In conclusion, our longitudinal analysis of renal and hematological function in NET patients treated with ^177^Lu-DOTATATE or ^90^Y-DOTATOC found no apparent renal toxicity. However, minor impairment of hematological function was observed. In addition, treatment with ^177^Lu-DOTATATE resulted in improved progression-free survival but did not significantly improve overall survival compared to ^90^Y-DOTATOC. PRRT with ^177^Lu-DOTATATE is safe with few side effects and can reduce the risk of disease progression. Future analyses should include a more generalizable study population, longer prospective follow-up, and implementation of dosimetry analysis to fully comprehend the toxicity profile and survival of PRRT in high-risk groups.

## Electronic Supplementary Material

Below is the link to the electronic supplementary material.

Supplementary Material 1

## Data Availability

The datasets generated during and/or analyzed during the current study are available from the corresponding author on reasonable request.
