# Supplementary material for: Peptide Receptor Radionuclide Therapy and clinical associations with renal and hematological toxicities and survival in patients with neuroendocrine tumors: an analysis from two U.S. medical centers
Source: J Cancer Res Clin Oncol. 2024 Nov 2;150(11):485. doi: 10.1007/s00432-024-06020-w (PMC11531437; doi:10.1007/s00432-024-06020-w)
Supplement: Supplementary file 1 — Supplementary Material 1 [file 432_2024_6020_MOESM1_ESM.docx]

Supplementary Materials

[**Supplementary Table 1.** Longitudinal changes in repeated measurements of renal function and association with type of PRRT 2](#_Toc180499585)

[**Supplementary Table 2.** Longitudinal changes in repeated measurements of hematological function and association with type of PRRT 3](#_Toc180499586)

[**Supplementary Table 3.** Longitudinal changes in odds of having grade 2 or 3 renal and hematological toxicities according to CTCAE v5.0 4](#_Toc180499587)

[**Supplementary Table 4.** Adjusted Hazard Ratios for Patients Who Received PRRT 5](#_Toc180499588)

[**Supplementary Table 5.** Subgroup analysis of chemotherapy prior to PRRT (bivariate analysis) 6](#_Toc180499589)

[**Supplementary Table 6.** ^177^Lu-DOTATATE sub-cohort in UIHC for changes in hematological function 7](#_Toc180499590)

[**Supplementary Table 7.** List of included chemotherapy targeted therapy 8](#_Toc180499591)

[**Supplementary Fig. 1** Flow diagram of case selection. 11](#_Toc180499592)

[**Supplementary Fig. 2** Kaplan-Meier curves of OS in UIHC patients who received ^177^Lu-DOTATATE and post-PRRT chemotherapy or targeted therapy. 12](#_Toc180499593)

# **Supplementary Table 1.** Longitudinal changes in repeated measurements of renal function and association with type of PRRT

|  | **N** | **Creatinine**  **β_-adjusted_ (95% CI)** | **P** | **BUN**  **β-_adjusted_ (95% CI)** | **P** | **eGFR (<60)**  **Odds ratio (95% CI)** | **P** |
| --- | --- | --- | --- | --- | --- | --- | --- |
| **Time 1** * **(months)** | - | -0.002 (-0.01, 0.00) | 0.54 | 0.01 (-0.10, 0.11) | 0.88 | **1.11 (1.03, 1.19)** | **0.01** |
| **Time 2 ^†^ (months)** | - | 0.01 (-0.004, 0.02) | 0.26 | 0.04 (-0.24, 0.32) | 0.79 | **0.88 (0.79, 0.97)** | **0.01** |
| **Type of PRRT** |  |  |  |  |  |  |  |
| ^177^Lu-DOTATATE  (vs ^90^Y-DOTATOC) | 335 | 0.11 (-0.09, 0.31) | 0.30 | -0.51 (-2.74, 1.71) | 0.65 | 0.32 (0.04, 2.58) | 0.29 |
| **Time 1** * **× ^177^Lu-DOTATATE (vs ^90^Y-DOTATOC)** | - | 0.004 (-0.003, 0.01) | 0.29 | -0.02 (-0.14, 0.10) | 0.78 | 0.94 (0.87, 1.00) | 0.07 |
| **Time 2 ^†^ × ^177^Lu-DOTATATE (vs ^90^Y-DOTATOC)** | - | -0.002 (-0.01, 0.01) | 0.72 | 0.17 (-0.15, 0.48) | 0.30 | 1.09 (0.99, 1.21) | 0.08 |
| **Time between date of diagnosis and initiation of PRRT (years)** | - | **0.02 (0.01, 0.03)** | **0.001** | **0.29 (0.17, 0.42)** | **<0.001** | **1.41 (1.23, 1.63)** | **<0.001** |
| **Age at diagnosis (years)** | - | 0.005 (-0.0003, 0.01) | 0.07 | **0.13 (0.08, 0.19)** | **<0.001** | **1.12 (1.05, 1.19)** | **<0.001** |
| **Sex at birth** |  |  |  |  |  |  |  |
| Female (vs Male) | 198 | **-0.25 (-0.37, -0.13)** | **<0.001** | **-1.38 (-2.69, -0.06)** | **0.04** | 0.64 (0.15, 2.63) | 0.53 |
| **Race/ethnicity** |  |  |  |  |  |  |  |
| Non-Hispanic White | 392 | [Reference] |  | [Reference] |  | [Reference] |  |
| Non-Hispanic Black | 37 | 0.15 (-0.07, 0.37) | 0.19 | 1.46 (-0.97, 3.88) | 0.24 | 0.25 (0.01, 4.72) | 0.35 |
| Other | 19 | 0.25 (-0.04, 0.55) | 0.09 | 2.33 (-0.96, 5.63) | 0.16 | 0.57 (0.02, 15.72) | 0.74 |
| **Primary tumor site** |  |  |  |  |  |  |  |
| GI tract (vs all other sites) | 242 | -0.13 (-0.32, 0.06) | 0.18 | **-3.00 (-5.12, -0.87)** | **0.01** | 0.49 (0.05, 4.47) | 0.53 |
| Pancreas (vs all other sites) | 106 | -0.16 (-0.36, 0.04) | 0.13 | **-3.00 (-5.30, -0.70)** | **0.01** | 0.24 (0.02, 2.87) | 0.26 |
| Lung (vs all other sites) | 31 | -0.19 (-0.46, 0.09) | 0.18 | -1.96 (-5.02, 1.10) | 0.21 | 0.08 (0.003, 2.45) |  |
| **Upfront chemotherapy or targeted therapy (Yes)** | 181 | 0.02 (-0.10, 0.15) | 0.73 | 0.75 (-0.65, 2.15) | 0.29 | 4.73 (0.95, 23.60) | 0.15 |
| **Primary tumor resection (Yes)** | 334 | 0.04 (-0.12, 0.20) | 0.64 | 0.92 (-0.85, 2.69) | 0.31 | 1.91 (0.28, 13.08) | 0.06 |
| **Count of multimorbidity** |  |  |  |  |  |  |  |
| 0 conditions | 272 | [Reference] |  | [Reference] |  | [Reference] |  |
| 1 - 2 conditions | 91 | 0.08 (-0.06, 0.23) | 0.27 | **2.05 (0.39, 3.70)** | **0.02** | 1.03 (0.18, 6.01) | 0.98 |
| 3 or more conditions | 14 | 0.26 (-0.02, 0.54) | 0.06 | **3.47 (0.36, 6.58)** | **0.03** | 2.39 (0.09, 60.21) | 0.60 |
| **Number of PRRT cycles** |  |  |  |  |  |  |  |
| 3-4 cycles (vs <3 cycles) | 318 | -0.07 (-0.21, 0.07) | 0.34 | 0.06 (-1.61, 1.73) | 0.94 | **0.12 (0.02, 0.72)** | **0.02** |
| **Liver directed therapy prior to PRRT (Yes)** | 184 | -0.07 (-0.19, 0.06) | 0.29 | -1.28 (-2.65, 0.09) | 0.07 | **0.12 (0.02, 0.56)** | **0.01** |
| **Institution** |  |  |  |  |  |  |  |
| LSU (vs UIHC) | 158 | 0.01 (-0.14, 0.17) | 0.86 | 0.65 (-1.04, 2.33) | 0.45 | **13.22 (1.75, 99.99)** | **0.01** |
| * Time 1 represents the follow-up time prior to and during PRRT.  † Time 2 represents the follow-up time after the last cycle of PRRT.  Age at diagnosis, type of PRRT, Time 1, Time2, Time 1 × type of PRRT, Time 2 × type of PRRT, race/ethnicity, sex at birth, primary tumor site, primary tumor resection, upfront chemotherapy or targeted therapy, count of comorbid conditions, number of PRRT cycles, time between date of diagnosis and initiation of PRRT, bone metastases, reduced doses and institution were included in models. | | | | | | | |

# **Supplementary Table 2.** Longitudinal changes in repeated measurements of hematological function and association with type of PRRT

|  | **N** | **WBC**  **β_-adjusted_ (95% CI)** | **P** | **Platelet counts**  **β-_adjusted_ (95% CI)** | **P** | **Hemoglobin**  **β-_adjusted_ (95% CI)** | **P** |
| --- | --- | --- | --- | --- | --- | --- | --- |
| **Time 1** * **(months)** | - | -0.02 (-0.06, 0.02) | 0.37 | -0.25 (-1.37, 0.87) | 0.66 | -0.01 (-0.04, 0.01) | 0.34 |
| **Time 2 ^†^ (months)** | - | 0.04 (-0.02, 0.10) | 0.18 | 0.10 (-1.39, 1.58) | 0.90 | -0.02 (-0.06, 0.03) | 0.45 |
| **Type of PRRT** |  |  |  |  |  |  |  |
| ^177^Lu-DOTATATE  (vs ^90^Y-DOTATOC) | 335 | -0.25 (-0.92, 0.42) | 0.47 | -19.47 (-51.13, 12.20) | 0.23 | 0.02 (-0.58, 0.62) | 0.94 |
| **Time 1** * **× ^177^Lu-DOTATATE (vs ^90^Y-DOTATOC)** | - | **-0.10 (-0.15, -0.04)** | **<0.001** | **-2.46 (-3.72, -1.19)** | **<0.001** | -0.03 (-0.06, 0.001) | 0.06 |
| **Time 2 ^†^ × ^177^Lu-DOTATATE (vs ^90^Y-DOTATOC)** | - | **0.09 (0.02, 0.16)** | **0.01** | **2.58 (0.89, 4.27)** | **0.003** | 0.03 (-0.02, 0.08) | 0.19 |
| **Time between date of diagnosis and initiation of PRRT (years)** | - | 0.004 (-0.03, 0.04) | 0.83 | **-1.84 (-3.53, -0.16)** | **0.03** | **-0.06 (-0.09, -0.03)** | **<0.001** |
| **Age at diagnosis (years)** | - | -0.01 (-0.03, 0.005) | 0.15 | **-1.07 (-1.81, -0.33)** | **0.005** | **-0.02 (-0.04, -0.01)** | **0.003** |
| **Sex at birth** |  |  |  |  |  |  |  |
| Female (vs Male) | 198 | -0.32 (-0.71, 0.08) | 0.12 | 17.89 (-0.44, 36.21) | 0.06 | **-0.82 (-1.16, -0.48)** | **<0.001** |
| **Race/ethnicity** |  |  |  |  |  |  |  |
| Non-Hispanic White | 392 | [Reference] |  | [Reference] |  | [Reference] |  |
| Non-Hispanic Black | 37 | -0.60 (-2.05, -0.10) | 0.13 | 3.39 (-29.67, 36.46) | 0.84 | **-1.20 (-1.82, -0.57)** | **<0.001** |
| Other | 19 | **-1.08 (-1.37, 0.17)** | **0.03** | -25.90 (-70.37, 18.56) | 0.25 | **-1.21 (-2.04, -0.38)** | **0.004** |
| **Primary tumor site** |  |  |  |  |  |  |  |
| GI tract (vs all other sites) | 242 | 0.11 (-0.55, 0.77) | 0.74 | -19.08 (-49.66, 11.50) | 0.22 | -0.15 (-0.72, 0.42) | 0.61 |
| Pancreas (vs all other sites) | 106 | **1.35 (0.63, 2.06)** | **<0.001** | 31.99 (-1.31, 65.30) | 0.06 | -0.03 (-0.65, 0.60) | 0.94 |
| Lung (vs all other sites) | 31 | **1.03 (0.10, 1.95)** | **0.03** | 5.70 (-36.47, 47.88) | 0.79 | 0.25 (-0.54, 1.04) | 0.54 |
| **Upfront chemotherapy or targeted therapy (Yes)** | 181 | -0.14 (-0.56, 0.28) | 0.52 | **-24.00 (-43.53, -4.47)** | **0.02** | **-0.54 (-0.90, -0.17)** | **0.004** |
| **Primary tumor resection (Yes)** | 334 | 0.28 (-0.26, 0.83) | 0.30 | **26.34 (1.87, 50.80)** | **0.03** | -0.09 (-0.55, 0.37) | 0.71 |
| **Count of multimorbidity** |  |  |  |  |  |  |  |
| 0 conditions | 272 | [Reference] |  | [Reference] |  | [Reference] |  |
| 1 - 2 conditions | 91 | 0.31 (-0.19, 0.82) | 0.23 | -6.21 (-29.79, 17.37) | 0.61 | -0.15 (-0.59, 0.29) | 0.51 |
| 3 or more conditions | 14 | 0.45 (-0.47, 1.37) | 0.34 | -12.94 (-59.31, 33.44) | 0.58 | **-1.31 (-2.16, -0.45)** | **0.003** |
| **Number of PRRT cycles** |  |  |  |  |  |  |  |
| 3-4 cycles (vs <3 cycles) | 318 | -0.48 (-1.00, 0.05) | 0.07 | -9.40 (-34.46, 15.66) | 0.46 | 0.01 (-0.47, 0.49) | 0.97 |
| **Liver directed therapy prior to PRRT (Yes)** | 184 | -0.09 (-0.50, 0.33) | 0.69 | -4.32 (-23.35, 14.72) | 0.66 | -0.02 (-0.37, 0.34) | 0.93 |
| **Bone metastases (Yes)** | 122 | -0.32 (-0.77, 0.12) | 0.15 | -7.26 (-27.22, 12.70) | 0.48 | -0.18 (-0.55, 0.20) | 0.35 |
| **Institution** |  |  |  |  |  |  |  |
| LSU (vs UIHC) | 158 | -0.40 (-0.91, 0.11) | 0.13 | -16.69 (-39.11, 5.74) | 0.14 | 0.18 (-0.24, 0.60) | 0.40 |
| ^*^ Time 1 represents the follow-up time prior to and during PRRT.  ^†^ Time 2 represents the follow-up time after the last cycle of PRRT.  Age at diagnosis, type of PRRT, Time 1, Time2, Time 1 × type of PRRT, Time 2 × type of PRRT, race/ethnicity, sex at birth, primary tumor site, primary tumor resection, upfront chemotherapy or targeted therapy, count of comorbid conditions, number of PRRT cycles, time between date of diagnosis and initiation of PRRT, bone metastases and institution were included in models. | | | | | | | |

**Supplementary Table 3.** Longitudinal changes in odds of having grade 2 or 3 renal and hematological toxicities according to CTCAE v5.0

| **CTCAE Term** | **Creatinine Increase**  **Odds ratio (95% CI)** | **WBC Decreased**  **Odds ratio (95% CI)** | **Platelet Count Decreased**  **Odds ratio (95% CI)** | **Anemia (Hemoglobin Decreased)**  **Odds ratio (95% CI)** |
| --- | --- | --- | --- | --- |
| **Monthly change of odds in grade 2 or 3 toxicities prior to and during PRRT in patients who received ^90^Y-DOTATOC** | 1.09 (0.98, 1.21) | 1.03 (1.00, 1.06) | 176.15  (3.44E-52, 9.01E+55) | **1.3 (1.06, 1.61)^*^** |
| **Monthly change of odds in grade 2 or 3 toxicities prior to and during PRRT in patients who received ^177^Lu-DOTATATE** | 1.12 (0.95, 1.31) | **1.07 (1.01, 1.13)^*^** | 1.17 (0.96, 1.42) | **1.18 (1.06, 1.32)^**^** |
| **Monthly change of odds in grade 2 or 3 toxicities after last cycle of PRRT in patients who received ^90^Y-DOTATOC** | 0.85 (0.69, 1.03) | 0.97 (0.92, 1.02) | 0.93 (0.69, 1.25) | **0.80 (0.65, 0.99)^*^** |
| **Monthly change of odds in grade 2 or 3 toxicities after last cycle of PRRT in patients who received ^177^Lu-DOTATATE** | 0.98 (0.88, 1.08) | 0.98 (0.94, 1.02) | 0.86 (0.72, 1.03) | **0.90 (0.81, 0.99) ^*^** |
| **Comparison of odds change in grade 2 or 3 toxicities prior to and during PRRT between ^177^Lu-DOTATATE and ^90^Y-DOTATOC** | 1.03 (0.87, 1.22) | 1.04 (0.98, 1.11) | 0.01  (1.30E-56, 3.38E+51) | 0.91 (0.74, 1.12) |
| **Comparison of odds change in grade 2 or 3 toxicities after last cycle of PRRT between ^177^Lu-DOTATATE and ^90^Y-DOTATOC (addition of two interaction terms)** | 1.15 (0.97, 1.38) | 1.01 (0.94, 1.08) | 0.93 (0.66, 1.29) | 1.12 (0.92, 1.36) |
| **Time between date of diagnosis and initiation of PRRT (years)** | 1.12 (0.98, 1.28) | 0.95 (0.88, 1.03) | 0.96 (0.81, 1.15) | 1.09 (1.00, 1.19) |
| *p < 0.05; **p < 0.01; ***p < 0.001  Age at diagnosis, type of PRRT, Time 1, Time2, Time 1 × type of PRRT, Time 2 × type of PRRT, race/ethnicity, sex at birth, primary tumor site, primary tumor resection, upfront chemotherapy or targeted therapy, count of comorbid conditions, number of PRRT cycles, time between date of diagnosis and initiation of PRRT, bone metastases (only for hematological function) and institution were included in models.  Grade 2 or 3 toxicities definition in CTCAE v5.0: Creatinine increased (creatinine >1.5 - 3.0 x baseline; >1.5 - 3.0 x ULN); WBC decreased (WBC <3000 - 2000/mm3; <3.0 - 2.0 x 10e9 /L); Platelet count decreased (platelet counts: <75,000 - 50,000/mm3; <75.0 - 50.0 x 10e9 /L); Anemia (hemoglobin <10.0 - 8.0 g/dL) | | | | |

# **Supplementary Table 4.** Adjusted Hazard Ratios for Patients Who Received PRRT

|  | **Progression (N)** | **Death**  **(N)** | **Overall Survival** | | **Progression-free Survival** | |
| --- | --- | --- | --- | --- | --- | --- |
|  |  |  | **HR (95%CI)** | **P** | **HR (95%CI)** | **P** |
| **Type of PRRT** |  |  |  |  |  |  |
| ^90^Y-DOTATOC | 56 | 81 | 1 [Reference] |  | 1 [Reference] |  |
| ^177^Lu-DOTATATE | 126 | 140 | 0.67 (0.40, 1.12) | 0.13 | **0.45 (0.26, 0.76)** | **0.003** |
| **Age at diagnosis (years)** | - | - | **1.03 (1.01, 1.04)** | **<0.001** | 1.01 (0.99, 1.02) | 0.40 |
| **Race** |  |  |  |  |  |  |
| Non-Hispanic White | 162 | 193 | 1 [Reference] |  | 1 [Reference] |  |
| Non-Hispanic Black | 15 | 19 | 1.11 (0.61, 2.04) | 0.73 | 1.24 (0.65, 2.37) | 0.51 |
| Other | 5 | 9 | 2.10 (0.92, 4.76) | 0.08 | 1.91 (0.74, 4.91) | 0.18 |
| **Sex at birth** |  |  |  |  |  |  |
| Male | 100 | 120 | 1 [Reference] |  | 1 [Reference] |  |
| Female | 81 | 101 | 1.04 (0.74, 1.46) | 0.82 | 1.07 (0.74, 1.55) | 0.73 |
| **Primary tumor site** |  |  |  |  |  |  |
| GI tract (vs all other sites) | 92 | 117 | 0.85 (0.49, 1.47) | 0.56 | 1.10 (0.55, 2.19) | 0.80 |
| Pancreas (vs all other sites) | 48 | 54 | 0.76 (0.42, 1.36) | 0.35 | 1.41 (0.71, 2.80) | 0.33 |
| Lung (vs all other sites) | 18 | 14 | **0.39 (0.16, 0.93)** | **0.03** | 1.33 (0.61, 2.92) | 0.48 |
| **Upfront chemotherapy or targeted therapy (Yes)** | 76 | 92 | **1.75 (1.17, 2.61)** | **0.01** | 1.26 (0.84, 1.87) | 0.27 |
| **WHO grade** |  |  |  |  |  |  |
| G1 | 38 | 42 | 1 [Reference] |  | 1 [Reference] |  |
| G2 | 81 | 86 | 0.83 (0.53, 1.28) | 0.40 | 1.10 (0.70, 1.73) | 0.67 |
| G3 | 16 | 20 | 1.37 (0.72, 2.60) | 0.34 | 1.52 (0.77, 3.00) | 0.22 |
| Unknown | 47 | 73 | 0.56 (0.32, 1.00) | 0.05 | 0.77 (0.41, 1.45) | 0.42 |
| **Liver metastases (Yes)** | 169 | 202 | 1.68 (0.85, 3.33) | 0.14 | **2.44 (1.14, 5.25)** | **0.02** |
| **Lymph nodes metastases (Yes)** | 118 | 124 | 0.83 (0.55, 1.26) | 0.38 | 0.85 (0.53, 1.37) | 0.51 |
| **Bone metastases (Yes)** | 62 | 66 | 1.00 (0.69, 1.45) | 0.99 | 1.31 (0.88, 1.94) | 0.19 |
| **Liver direct therapy prior to PRRT (Yes)** | 82 | 101 | 1.15 (0.79, 1.66) | 0.47 | 1.35 (0.92, 1.97) | 0.13 |
| **Primary tumor resection (Yes)** | 137 | 153 | 0.70 (0.46, 1.08) | 0.10 | 0.81 (0.50, 1.32) | 0.40 |
| **Count of comorbid conditions** |  |  |  |  |  |  |
| 0 conditions | 109 | 112 | 1 [Reference] |  | 1 [Reference] |  |
| 1 or 2 conditions | 35 | 50 | 1.15 (0.75, 1.76) | 0.52 | 0.66 (0.41, 1.07) | 0.09 |
| 3 or more conditions | 7 | 8 | 1.41 (0.57, 3.52) | 0.46 | 0.89 (0.36, 2.23) | 0.81 |
| **Time between** **date of diagnosis and initiation of PRRT (years)** | - | - | 1.00 (0.96, 1.04) | 0.87 | 1.04 (1.00, 1.08) | 0.05 |
| **Number of PRRT doses received** |  |  |  |  |  |  |
| <3 cycles | 31 | 88 | 1 [Reference] |  | 1 [Reference] |  |
| 3-4 cycles | 151 | 132 | **0.31 (0.21, 0.47)** | **<0.001** | 1.14 (0.64, 2.03) | 0.65 |
| **Reduced radioactivity (Yes)** | 21 | 52 | 0.90 (0.52, 1.56) | 0.71 | 0.63 (0.35, 1.11) | 0.11 |
| **Institution** |  |  |  |  |  |  |
| UIHC | 126 | 153 | 1 [Reference] |  | 1 [Reference] |  |
| LSU | 56 | 68 | 1.44 (0.86, 2.41) | 0.16 | 0.78 (0.45, 1.33) | 0.36 |
| Age at diagnosis, type of PRRT, race/ethnicity, sex at birth, primary tumor site, primary tumor resection, chemotherapy prior to PRRT, count of comorbid conditions, number of PRRT cycles, time between date of diagnosis and initiation of PRRT, liver metastases, lymph node metastases, bone metastases, liver direct therapy prior to PRRT, reduced dose(s) and institution were included in models. | | | | | | |

# **Supplementary Table 5.** Subgroup analysis of chemotherapy prior to PRRT (bivariate analysis)

|  | **Previous Chemotherapy (Yes)**  **(N=181)** | **Previous Chemotherapy (No)**  **(N=267)** | **P** |
| --- | --- | --- | --- |
| **Age at diagnosis (years),** Mean (SD) | 53.06 (13.53) | 55.07 (13.79) | 0.13 |
| **Time between date of diagnosis and initiation of PRRT (years),** Mean (SD) | 6.04 (13.53) | 6.65 (5.51) | 0.79 |
| **Type of PRRT** |  |  | 0.06 |
| ^90^Y-DOTATOC | 37 (20.44) | 76 (28.46) |  |
| ^177^Lu-DOTATATE | 144 (79.56) | 191 (71.54) |  |
| **Race** |  |  | 0.11 |
| Non-Hispanic White | 156 (86.19) | 236 (88.39) |  |
| Non-Hispanic Black | 20 (11.05) | 17 (6.37) |  |
| Other | 5 (2.76) | 14 (5.24) |  |
| **Sex at birth** |  |  | 0.70 |
| Male | 99 (54.70) | 151 (56.55) |  |
| Female | 82 (45.30) | 116 (43.45) |  |
| **Primary tumor site** |  |  | **<0.001** |
| GI tract | 75 (41.44) | 167 (62.55) |  |
| Pancreas | 63 (34.81) | 43 (16.10) |  |
| Lung | 15 (8.29) | 16 (5.99) |  |
| **WHO grade** |  |  | **<0.001** |
| G1 | 38 (20.99) | 70 (26.22) |  |
| G2 | 84 (46.41) | 100 (37.45) |  |
| G3 | 22 (12.15) | 11 (4.12) |  |
| Unknown | 37 (20.44) | 86 (32.21) |  |
| **Liver metastases (Yes)** | 164 (90.61) | 238 (89.14) | 0.62 |
| **Lymph nodes metastases (Yes)** | 86 (47.78) | 186 (69.66) | **<0.001** |
| **Bone metastases (Yes)** | 57 (31.49) | 65 (24.34) | 0.10 |
| **Liver direct therapy prior to PRRT (Yes)** | 80 (45.71) | 104 (39.10) | 0.17 |
| **Primary tumor resection (Yes)** | 120 (66.30) | 214 (80.75) | **<0.001** |
| **Count of comorbid conditions** |  |  | 0.10 |
| 0 conditions | 123 (76.88) | 149 (68.66) |  |
| 1 or 2 conditions | 30 (18.75) | 61 (28.11) |  |
| 3 or more conditions | 7 (4.38) | 7 (3.23) |  |
| **Number of PRRT doses received** |  |  | 0.21 |
| <3 cycles | 46 (25.41) | 82 (30.94) |  |
| 3-4 cycles | 135 (74.59) | 183 (69.06) |  |
| **Reduced radioactivity(s) (Yes)** | 22 (12.36) | 30 (11.45) | 0.77 |
| **Institution** |  |  | **<0.001** |
| UIHC | 98 (54.14) | 192 (71.91) |  |
| LSU | 83 (45.86) | 75 (28.09) |  |

# **Supplementary Table 6.** ^177^Lu-DOTATATE sub-cohort in UIHC for changes in hematological function

|  | **WBC**  **β_-adjusted_ (95% CI)** | **P** | **Platelet counts**  **β-_adjusted_ (95% CI)** | **P** | **Hemoglobin**  **β-_adjusted_ (95% CI)** | **P** |
| --- | --- | --- | --- | --- | --- | --- |
| **Monthly rate of change in hematological function prior to and during PRRT in patients who received ^177^Lu-DOTATATE** | **-0.11**  **(-0.14, -0.08)** | **<0.001** | **-1.81**  **(-2.45, -1.17)** | **<0.001** | **-0.03**  **(-0.05, -0.01)** | **<0.001** |
| **Monthly rate of change in hematological function after last cycle of PRRT in patients who received ^177^Lu-DOTATATE** | 0.02  (-0.01, 0.04) | 0.18 | -0.05  (-0.58, 0.49) | 0.82 | **-0.02**  **(-0.04, -0.01)** | **0.001** |
| Age at diagnosis, Time 1, Time2, race/ethnicity, sex at birth, primary tumor site, primary tumor resection, upfront chemotherapy or targeted therapy, chemotherapy after PRRT, count of comorbid conditions, number of PRRT cycles, time between date of diagnosis and initiation of PRRT, bone metastases and institution were included in models. | | | | | | |

# **Supplementary Table 7.** List of included chemotherapy targeted therapy

| Carboplatin/Etoposide |
| --- |
| Cisplatin/Etoposide |
| Interferon |
| Capecitabine and Temozolomide (CAPTEM) |
| Temozolomide |
| FOLFOX |
| Pembrolizumab/axitinib |
| Everolimus (Afinitor) |
| FOLFIRINOX |
| Fluorouracil (5-FU) |
| Doxorubicin (Adriamycin) |
| Streptozocin |
| Sunitinib |
| PDR001 (clinical trial) |
| NET-001 clinical trial ABI-009 |
| FOLFIRI |
| PEN 221 (clinical trial) |
| Denosumab (XGEVA) |
| Capecitabine (Xeloda) |
| Nivolumab and Ipilimimumab |
| Sorafenib (Nexavar) |
| Dacarbazine (DTIC) |
| Bevacizumab (Avastin) |
| Pembrolizumab and defactinnib |
| Azacitidine |
| Paclitaxel (Abraxane) |
| VX-803 Refmal |
| CPTK787/ZK222584 |
| Cyclophosphamide (Cytoxan) |
| Doxorubicin (Lipodox) |
| Vincristine |
| Epothilone B |
| Docetaxel (Taxotere) |
| Thalidomide |
| VP-16 |
| Vandetanib |
| BOS172738 |
| carboplatin and paclitaxel |
| Pazopanib |


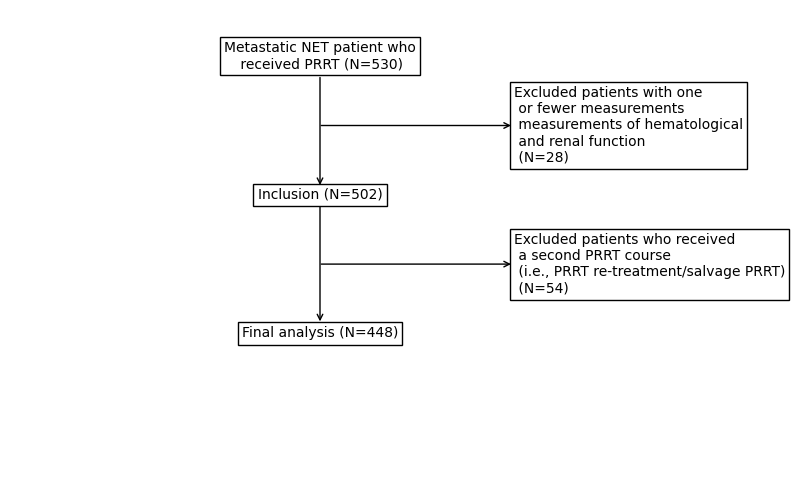


# **Supplementary Fig. 1** Flow diagram of case selection.


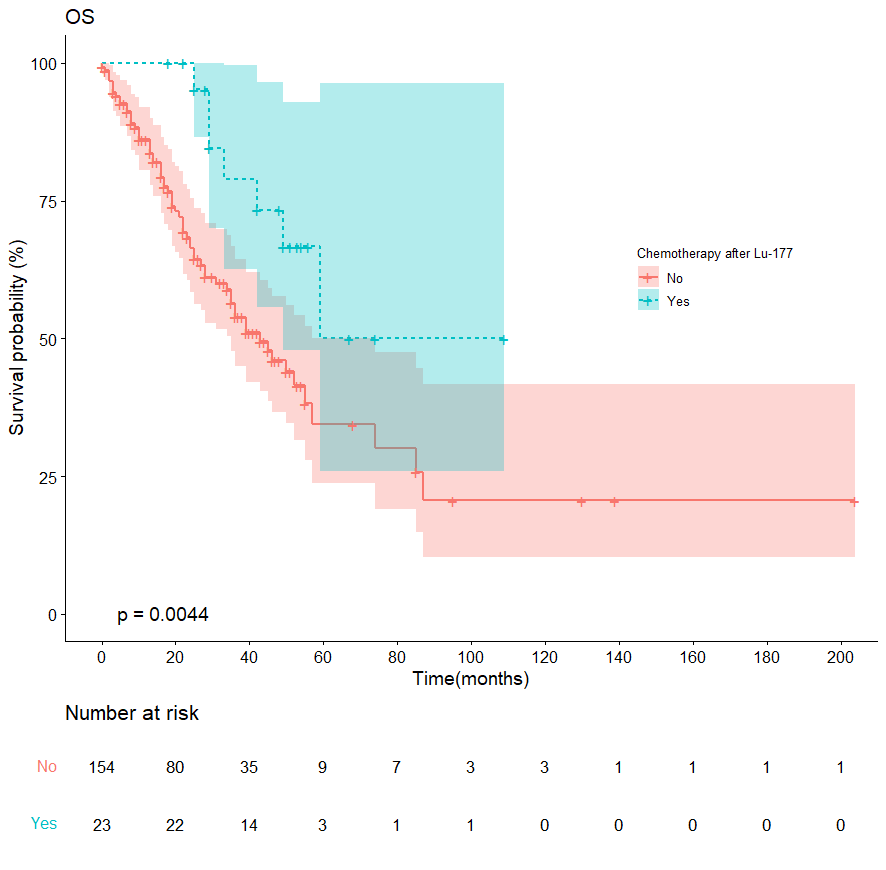


# **Supplementary Fig. 2** Kaplan-Meier curves of OS in UIHC patients who received ^177^Lu-DOTATATE and post-PRRT chemotherapy or targeted therapy.
